# IFNγ-Induced MHC Class II Expression on Islet Endothelial Cells Is an Early Marker of Insulitis but Is Not Required for Diabetogenic CD4^+^ T Cell Migration

**DOI:** 10.3389/fimmu.2018.02800

**Published:** 2018-11-28

**Authors:** Nicholas A. Scott, Yuxing Zhao, Balasubramanian Krishnamurthy, Stuart I. Mannering, Thomas W. H. Kay, Helen E. Thomas

**Affiliations:** ^1^St. Vincent's Institute, Fitzroy, VIC, Australia; ^2^Department of Medicine, St. Vincent's Hospital, The University of Melbourne, Fitzroy, VIC, Australia

**Keywords:** Type 1 diabetes, MHC class II, interferon-γ, endothelial cells, non-obese diabetic mice

## Abstract

Diabetogenic T cells infiltrate the pancreatic islets by transmigrating across the microcapillaries residing close to, or within, the pancreatic islets. Deficiency in IFNγ signaling prevents efficient migration of T cells into the pancreatic islets, but the IFNγ-regulated molecules that mediate this are uncertain. Homing of autoreactive T cells into target tissues may require antigen specificity through presentation of cognate antigen by MHC expressed on the vascular endothelium. We investigated the hypothesis that IFNγ promotes the migration of islet antigen-specific CD4^+^ T cells by upregulating MHC class II on islet endothelial cells (IEC), thereby providing an antigen-specific signal for islet infiltration. Upon IFNγ stimulation, MHC class II, which is not constitutively expressed on IEC, was induced. IFNγ-dependent upregulation of MHC class II was detected in IEC isolated from prediabetic NOD mice at the earliest stages of insulitis, before other markers of inflammation were present. Using a CD4^+^ T cell-mediated adoptive transfer model of autoimmune diabetes we observed that even though diabetes does not develop in recipient mice lacking IFNγ receptors, mice with MHC class II-deficient IEC were not protected from disease. Thus, IFNγ-regulated molecules, but not MHC class II or antigen presentation by IECs is required for the early migration of antigen-specific CD4^+^ T cells into the pancreatic islets.

## Introduction

A defining early step in the pathogenesis of type 1 diabetes (T1D) in mice and humans is the infiltration of the pancreatic islets with immune cells (termed insulitis), including beta-cell antigen specific T cells (1–3). Genetic studies of T1D in humans and mouse models indicate that susceptibility to autommune diabetes is strongly dependent on the MHC/HLA class II genes (4, 5), implicating CD4^+^ T cells. CD4^+^ T cells have consistently been identified as the first lymphocytes to occupy the islets in the NOD mouse (6–8), and beta cell-specific CD4^+^ T cells infiltrate human islets (9–11). It has been suggested that preventing this early seeding of the islets could be an effective form of therapy for preventing disease (12, 13).

Diabetogenic T cells are activated in the pancreatic lymph nodes (PLNs) (14), before traveling through the peripheral circulation to the pancreas and islets via microcapillaries. This process requires interaction between lymphocytes and endothelial cells (EC) lining the vessel wall. These interactions are mediated by endothelial cell adhesion molecules (CAMs) and integrins on T cells (1–3, 13). Prior to integrin-CAM engagement, there is evidence in autoimmune and allograft transplant models that T cells require a cognate antigen signal, presented by MHC class I or MHC class II expressed on EC, to enter tissues (4, 5, 15–19). In this hypothesis, EC residing in microvessels near the graft or autoimmune target acquire antigen from the microenvironment and present it into the lumen of the capillary to circulating T cells.

Interferon-gamma (IFNγ) has been implicated in the pathogenesis of autoimmune diabetes (6–8, 20, 21). Protection from diabetes mediated by T-cell transfer occurs when splenocytes from diabetic NOD mice are adoptively transferred into irradiated NOD.*Ifn*γ*r2*^−/−^ recipient mice, but not vice versa (22). Examination of the islets of protected mice demonstrated that IFNγR deficiency significantly reduced T cell migration into the islets. As protection is only seen in NOD.*Ifn*γ*r2*^−/−^ and not NOD recipients, this implies IFNγ is acting at least in part on a non-immune cell. Disruption of IFNγR specifically on beta cells had no effect on diabetes (12, 13, 23), raising the possibility that endothelial cells residing in the islet (islet endothelial cells, IEC) are actively involved in T cell infiltration into islets. IECs express IFNγR, and when challenged with IFNγ may permit T cells to leave the circulation and enter tissues (13, 14, 22). EC express MHC class I constitutively, but under inflammatory conditions cytokines such as IFNγ can upregulate MHC class I and induce the expression of HLA/MHC class II in human (24–26) and murine (27) EC lines.

The question we are addressing here is whether migration into islets involves presentation of antigen to CD4^+^ T cells by class II MHC proteins on EC or not. We have also investigated whether IFNγ promotes upregulation of MHC class II on IECs *in vitro*, and examined MHC class II expression on IECs during progression to diabetes in NOD mice.

## Materials and methods

### Mice

All mice were bred and maintained under specific pathogen free (SPF) conditions at St Vincent's Institute and were approved by the St Vincent's Hospital Animal Ethics Committee. IFNγR2-deficient (28) and NOD.Rag1-deficient (29) mice on a NOD/Lt background were purchased from the Type I Diabetes Repository at the Jackson Laboratories (Bar Harbor, ME, USA). NOD.BDC2.5 TCR transgenic mice (30), which express a CD4^+^ T-cell-associated TCR transgene specific for I-A^g7^ restricted β-cell-antigen chromogranin A, were provided by D. Mathis and C. Benoist (Harvard University, MA, USA). NOD.MHC cII^−/−^ mice (NOD.*H2-Ab1*^−/−^) (31) lack all conventional MHC class II genes and were provided by J. McCluskey (University of Melbourne, Australia). Wild-type, age, and sex-matched NOD mice, co-housed with knockout or transgenic mice, were used as controls for experiments.

### Islet isolation and culture

Islets of Langerhans were isolated from mice using collagenase P (Roche) and histopaque-1077 density gradients (Sigma-Aldrich) as described previously (32). Isolated islets were either made into single cell suspensions for flow cytometry analysis or handed picked for IFNγ stimulation studies. To make a single cell suspension, islets were trypsinised with bovine trypsin (342 U/mL, Calbiochem) and 2 mM EDTA in PBS, washed to remove trypsin and rested in culture medium at 37°C for 1.5 h. For stimulation studies, islets were hand picked and cultured in CMRL medium-1066 (Life Technologies), containing penicillin/streptomycin, 2 mM glutamine, and 10% FCS. Mouse islets were cultured with or without 1 ng/mL of murine IFNγ (R&D systems) in CMRL medium-1066 overnight as described (23).

### Flow cytometry

Antibodies used (with clone name and manufacturer in brackets) were anti-CD45 conjugated to PerCP-Cy5.5 (30-F11, Biolegend), Anti-I-A^g7^ (OX6, BD Pharmingen) conjugated to APC, pan endothelial antigen (MECA-32, Biolegend) conjugated to Alexa-488, anti-CD31 (MEC-13.3, BD Pharmingen) conjugated to PE, anti-CD25 (PC61, BD Biosciences) conjugated to APC, clonotypic BDC2.5 TCR (provided by Dr O Kanagawa) (33) and anti-mouse Alexa-488 (Life Technologies). Where PerCP-Cy5.5 was used, propidium iodine (Calbiochem) was added (1 μg/mL) to exclude dead cells. Beta cells were differentiated from other islet cells by autoflorescence (FITC channel). Analysis was performed using a FACS Fortessa (BD Biosciences) and FlowJo analysis software (Tree Star, Inc.). Gating strategies are shown in the figures.

### Adoptive T-cell transfer

Cell suspensions of spleen and PLN from NOD.BDC2.5 mice were stained with anti-CD4 MicroBeads (L3T4, Miltenyi Biotech) and magnetically separated on an autoMACS according to manufacturer's instructions (Miltenyi Biotech). CD4^+^ cells were stained with anti-CD25 and clonotypic BDC2.5 TCR and the BDC2.5^high^ CD25^−^ population was sorted on a FACS Aria (BD Biosciences). Cells were washed, counted and injected intravenously into sublethally irradiated (750 rads) recipient mice (5 × 10^5^ T cells/mouse). For diabetes monitoring, mice with blood glucose readings of ≥15 mM/L were considered diabetic.

### Histology

Pancreata were snap frozen in OCT (Sakura Finetek) and 5 μm sections prepared. Slides were stained with guinea pig anti-insulin polyclonal Ab (Dako Cytomation), followed by horseradish peroxidase-conjugated anti-guinea pig Ig (Dako Cytomation) as described previously (23).

### Bone marrow chimeras

Femurs and tibias from NOD.*Rag1*^−/−^ mice were flushed using a 26G needle, mashed through a cell strainer, washed and counted. 5 × 10^6^ cells were injected intravenously into lethally irradiated (two doses of 550 rad) NOD.*Rag1*^−/−^ or NOD.*Rag1*^−/−^MHC cII^−/−^ recipient mice. After allowing 8 weeks for reconstitution, mice were adoptively transferred with 5 × 10^5^ CD4^+^BDC2.5^high^CD25^−^ T cells. Blood glucose was measured every day from day 7 post transfer. Mice with blood glucose of ≥15 mM/L were considered diabetic.

### Statistics

All statistical analysis was performed using Prism 7 (GraphPad Software). Diabetes incidence was assessed by a log-rank test (survival curve analysis). Correlations were calculated using a two-tailed Pearson correlation coefficient and a line of best fit was placed using the linear regression function within Prism 7.

## Results

### MECA-32 is a specific marker of endothelial cells in pancreatic islets and is maintained during insulitis

**Figure 1:**
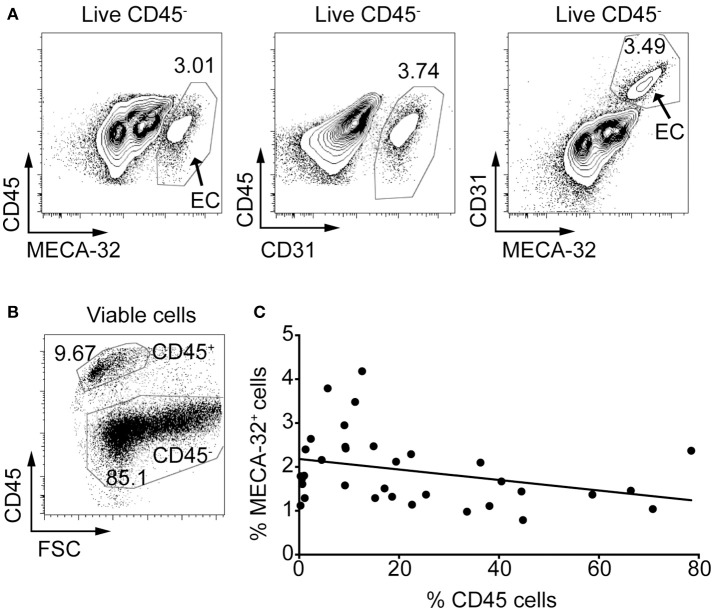
Anti-MECA-32 antibody identifies endothelial cells in the pancreatic islets, even in late stages of insulitis. Islets were isolated from NOD mice and trypsinized to a single cell suspension. Cells were stained with antibodies to CD45, propidium iodide (PI) and the endothelial cell markers, CD31 and MECA-32, and analyzed by flow cytometry. **(A)** Ten-week old NOD mouse islets stained with CD31 and MECA-32 (left and middle panels, respectively) and double stained (right panel). Representative plots from one mouse shown (from *n* = 5 mice). **(B)** Gating strategy for immune cells (CD45^+^) and islet cells (CD45^−^). **(C)** Islets from NOD mice aged 4–22 weeks were isolated and analyzed as above. Percentage CD45^+^ cells in the islets was compared to the percentage of MECA-32 endothelial cells for each individual mouse and plotted. *N* = 34 mice, *r* = −0.3249, *R*^2^ = 0.1056, *p* = 0.0608 linear regression.

The use of flow cytometry is an ideal method to examine MHC class II expression in islet endothelial cells because the use of stringent gating strategies can eliminate the possibility of detecting other cell types such as leukocytes. First, we examined whether two known EC markers, CD31 (also known as platelet endothelial cell adhesion molecule, PECAM-1) and pan-endothelial antigen (MECA-32) could be used for identification of endothelial cells in the islets during insulitis. Although CD31 is often used to identify vasculature by histology and flow cytometry, it can also be found on other cells, including macrophages, granulocytes, and lymphocytes, among others. MECA-32 on the other hand is restricted to ECs (34). Islets were isolated from 10-week-old NOD mice, trypsinised to obtain single-cell suspensions, stained with antibodies to CD45 (to identify leukocytes), CD31 and MECA-32, and analyzed by flow cytometry. MECA-32 and CD31 stained approximately the same proportion of CD45^−^ cells in the islet and co-stained the same cells (Figure 1A).

We investigated whether mice with heavily infiltrated islets (defined as islet preparations containing >30% CD45^+^ cells) lose their IECs due to disruption of the islet structure when beta cells are specifically destroyed. The proportion of CD45^+^ cells was used as a marker of immune infiltration (Figure 1B). The percentage of CD45^+^ cells was compared to the proportion of IECs (%MECA-32^+^CD45^−^) in islet preparations from individual NOD mice (Figure 1C). The percentage of MECA-32^+^ cells in mice varied between 0.8 and 4.2% of total islet cells (Figure 1C), consistent with previous findings (35). While there was a trend toward a reduction in MECA-32^+^ cells with increasing CD45^+^ cells, this was not statistically significant; IECs were still identifiable even in heavily infiltrated islets. This observation implies that IECs and microvessels within the islets are largely maintained as insulitis proceeds.

### IFNγ upregulates MHC class II on islet endothelial cells *in vitro*

**Figure 2:**
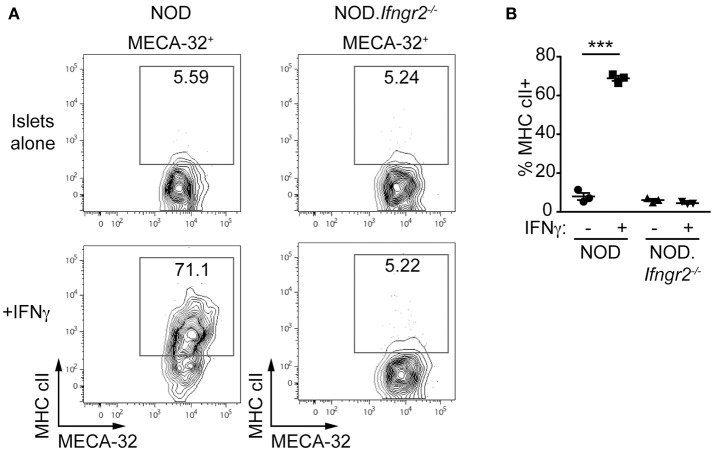
Recombinant IFNγ induces MHC class II on NOD, but not NOD.Ifnγr2^−/−^, islet endothelial cells. Islets were isolated from 6 week NOD and NOD.*Ifn*γ*r2*^−/−^ mice and cultured overnight with or without 1 ng/ml IFNγ, then trypsinised into single-cell suspensions and stained for flow cytometry. **(A)** Representative FACS plots demonstrating expression of NOD MHC class II, I-A^g7^, on endothelial cells (MECA-32^+^) is shown for NOD and NOD.*Ifn*γ*r2*^−/−^. **(B)** Quantified data showing the proportion of MECA32^+^ MHC cII^+^ cells for *n* = 3 independent experiments, ^***^p < 0.0001, one-way ANOVA.

We examined MHC class II expression on IECs and whether IFNγ can induce expression of MHC class II on the islet vasculature. Islets from 6-week old NOD and NOD.*Ifn*γ*r2*^−/−^ mice were cultured with or without IFNγ and analyzed for MHC class II expression. CD45^+^ cells were not detected in any of the islet cell suspensions indicating the islets were isolated before onset of insulitis (data not shown). Addition of IFNγ lead to MHC class II upregulation on IECs in NOD islets, while in NOD.*Ifn*γ*r2*^−/−^ derived islets no MHC class II expression was induced (Figures 2A,B). These data suggest IFNγ directly induces MHC class II expression on ECs of the microvessels of the islet.

### MHC class II is upregulated on endothelial cells in the early stages of islet infiltration

**Figure 3:**
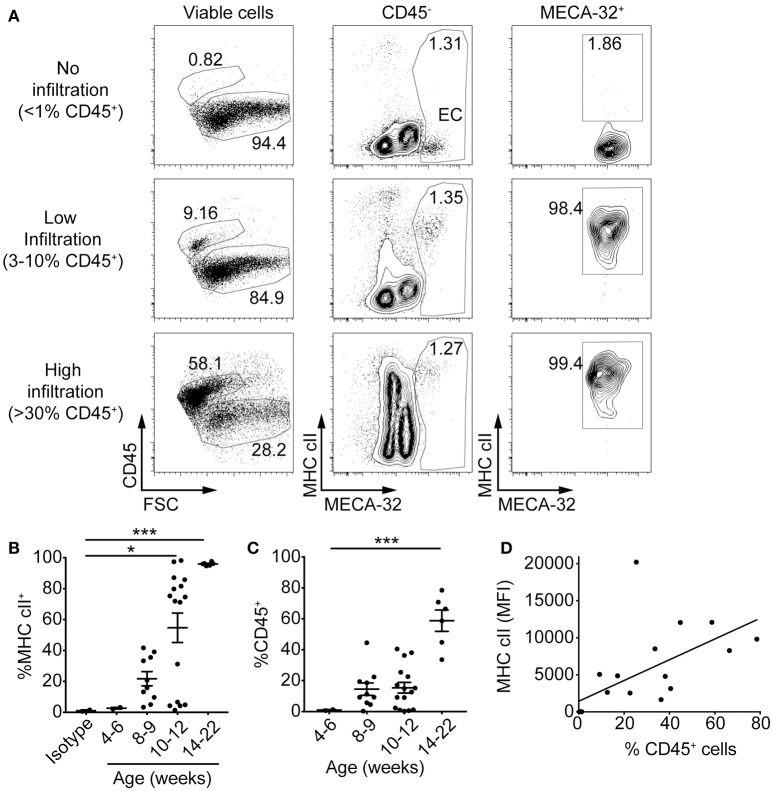
MHC class II on islet endothelial cells is upregulated in the early stages of islet infiltration in NOD mice. Islets were isolated from 4 to 22 week old NOD mice and single cells stained with antibodies to NOD MHC class II, I-A^g7^, CD45 for leukocytes, MECA-32 for endothelial cells, and propidium iodide (PI) for viability. **(A)** Representative plots of MHC class II expression on IEC from NOD islets with no infiltration (upper panel, as determined by < 1% live cells with CD45 staining), low (middle panel, 3–10% live cells CD45^+^) and high (lower panel, >30% live cells CD45^+^) levels of infiltration. **(B,C)** The percentage of **(B)** MHC class II-positive islet endothelial cells ^*^p = 0.04, ^***^p = 0.0008 (one-way ANOVA) and, **(C)** CD45^+^ cells for NOD mice at different ages ^***^p < 0.0001 (one-way ANOVA). Data combined from 9 separate experiments, 4–6 weeks (*n* = 2), 8–9 weeks (*n* = 10), 10–12 weeks (*n* = 16), 14–22 weeks (*n* = 6), mean±SEM. **(D)** The percentage of CD45^+^ cells within the islets was plotted against median fluorescence intensity (MFI) of MHC class II on IEC (MECA-32^+^) for each individual mouse. Data combined from five independent experiments, *n* = 19, *r* = 0.6155, *r*^2^ = 0.3788, *p* < 0.01, two-tailed linear regression.

If presentation of cognate antigen by IFNγ-induced MHC class II to diabetogenic T cells is a key process required for homing of the first CD4^+^ T cells into the islets, then upregulation of MHC class II on IECs should occur early. We isolated islets from 4 to 22-week old NOD mice with varying levels of insulitis. Islet cell suspensions were stained for MHC cII I-A^g7^, CD45 and MECA-32 and examined by flow cytometry. Examination of IECs for MHC class II expression in islets from young NOD mice with no infiltration (< 1% CD45^+^) showed no expression of MHC class II on MECA-32^+^ endothelial cells (Figure 3A). In contrast, islets from mice with a detectable but low proportion of CD45^+^ cells (3–10% CD45^+^) demonstrated strong expression of MHC class II on endothelial cells. Mice with an increased proportion of CD45^+^ cells (>30% CD45^+^) maintained high levels of MHC class II expression.

The percentage of IEC that were MHC class II^+^ increased with age (Figure 3B) and correlated with the proportion of CD45^+^ cells in the islets (Figure 3C). NOD mice at 4–6 weeks showed no IEC MHC class II expression. At 8 and 9 weeks MHC class II expression was evident in those mice that also had CD45^+^ cells. At 10–12 weeks, a majority of mice showed >75% MHC class II^+^ IECs, and those that did not also had no CD45^+^ cells. By 14–22 weeks, all mice had CD45^+^ cells and expression of MHC class II on their islet vasculature. There was a direct correlation between median fluorescent intensity of MHC class II on MECA-32^+^ cells and percentage of CD45^+^ cells (Figure 3D). The upregulation of MHC class II on IECs was observed in younger mice and in islets with less CD45^+^ cells than that observed on beta cells, where MHC class II was upregulated by 10–12 weeks of age in mice with >20% CD45^+^ cells in their islets (36). Together, these observations suggest first that MHC class II upregulation on the islet vasculature occurs around the same time as the first T cells infiltrate the islets, [and before MHC class II is expressed on beta cells (36)], and second that this upregulation is directly correlated with the infiltration of immune cells into the islets.

### MHC class II upregulation on islet endothelial cells does not occur in NOD.*ifnγr2*^−/−^ mice

**Figure 4:**
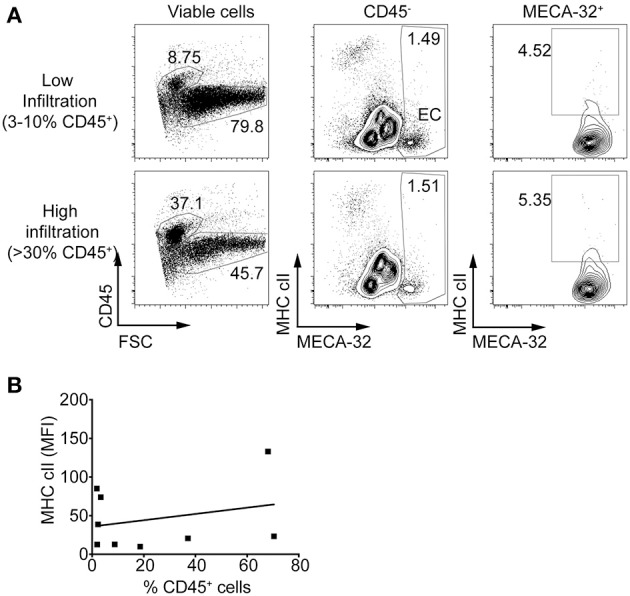
Islet endothelial cells isolated from NOD.Ifnγr2^−/−^ mice do not express MHC class II. Islets were isolated and single cells stained for MHC class II and MECA-32. **(A)** Representative plots of MHC class II expression on islets from NOD.*Ifn*γ*r2*^−/−^ mice showing low infiltration (upper panel, 3–10% live cells CD45^+^) and high infiltration (lower panel, >30% live cells CD45^+^). **(B)** The percentage of CD45^+^ cells within the islets was plotted against median fluorescence intensity of MHC class II on IEC for each individual mouse and correlated. *N* = 9, *r* = 0.2688, *r*^2^ = 0.07228, *p* = 0.4842 two-tailed linear regression.

We next determined if MHC class II expression on IECs *in vivo* is dependent on IFNγ. MHC class II upregulation was not observed on IECs in NOD.*Ifn*γ*r2*^−/−^ mice, even when insulitis was present (Figure 4A). In contrast to NOD islets (Fig. 3D) there was no relationship between the percentage of CD45^+^ cells in the islet and median fluorescence intensity of MHC class II on IEC in NOD.*Ifn*γ*r2*^−/−^ mice (Figure 4B). These data suggest MHC class II upregulation on the vasculature of the islets is dependent on IFNγ, but also implies MHC class II expression on IECs is not absolutely required for CD4^+^ and CD8^+^ T cells to infiltrate the islets.

### Chimeric NOD mice lacking MHC class II on endothelial cells develop diabetes

Previously it has been shown that there is reduced insulitis in the first 48 h post-adoptive transfer of activated BDC2.5 CD4^+^ diabetogenic T cells into NOD.*Rag1*^−/−^ mice treated with anti I-A^g7^ blocking antibody (37). Yet, administering anti-I-A^g7^ antibody could also block antigen presentation by APC, hence conclusions cannot be drawn on whether this effect is due to blockade of IEC-MHC class II. We therefore generated a model to determine whether MHC class II on IECs is required for diabetogenic CD4^+^ T cell homing, while maintaining MHC class II expression on immune cells.

**Figure 5:**
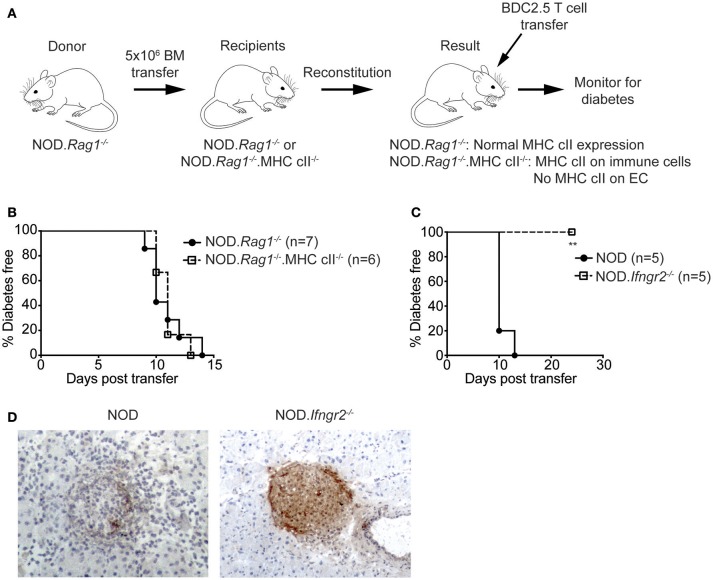
Protection from CD4^+^ T cell adoptive transfer diabetes in Ifnγr2-deficient NOD mice is not mediated through the inability of the islet vasculature to express MHC class II. **(A)** Experimental method: 5 × 10^6^ NOD.*Rag1*^−/−^ donor bone marrow was adoptively transferred into lethally irradiated NOD.*Rag1*^−/−^ or NOD.*Rag1*^−/−^.MHC cII^−/−^ recipient mice. After allowing 8 weeks for reconstitution, CD4^+^ BDC2.5^high^ CD25^−^ T cells were sorted from NOD.BDC2.5 spleens and PLNs, and 0.5 × 10^6^ cells were IV injected into recipient mice. **(B)** Diabetes incidence of NOD.*Rag1*^−/−^ (*n* = 7) or NOD.*Rag1*^−/−^.MHC cII^−/−^ (*n* = 6) chimeric recipient mice after adoptive transfer of CD4^+^ BDC2.5^high^ CD25^−^ T cells. *P* = 0.9456, log-rank test. **(C,D)** NOD and NOD.*Ifn*γ*r2*^−/−^ mice were IV injected with 0.5 × 10^6^ CD4^+^ BDC2.5^high^ CD25^−^ T cells and diabetes was monitored. **(C)** Diabetes incidence of NOD (*n* = 5) and NOD. *Ifn*γ*r2*^−/−^ (*n* = 5) after adoptive transfer of BDC2.5 T cells. ^**^p < 0.01 log-rank test. **(D)** Representative images of pancreatic sections stained with anti-insulin antibody from NOD and NOD.*Ifn*γ*r2*^−/−^ recipient mice. Magnification 200x.

We used a chimeric bone marrow reconstitution model. NOD.*Rag1*^−/−^ bone marrow was transplanted into lethally irradiated NOD.*Rag1*^−/−^ and NOD.*Rag1*^−/−^.MHCcII^−/−^ mice (Figure 5A). Resulting NOD.*Rag1*^−/−^ recipients had MHC class II expressed on immune cells and ECs, whereas NOD.*Rag1*^−/−^.MHC cII^−/−^ recipient mice had MHC class II only on immune cells and not on IECs (and other non-immune host cells). After allowing eight weeks for reconstitution, mice were injected with 5 × 10^5^ sorted CD4^+^ BDC2.5^high^ CD25^−^ T cells, and diabetes monitored. NOD recipient mice developed diabetes after 9–14 days (Figure 5B). NOD. *Rag1*^−/−^MHC cII^−/−^ mice were all diabetic by day 13. This result reveals that IECs do not require MHC class II to allow diabetes to occur in a CD4^+^ T cell adoptive transfer model. In contrast, diabetes was prevented when CD4^+^ BDC2.5^high^ CD25^−^ T cells were adoptively transferred into sublethally irradiated NOD.*Ifngr2*^−/−^ but not wild-type NOD recipients (Figure 5C). Pancreas sections from recipient mice showed immune infiltration and beta cell destruction in the NOD recipients, while most of the islets from NOD.*Ifngr2*^−/−^ recipients were still intact with no signs of insulitis (Figure 5D). Together these data suggest that the protection from diabetes seen in mice deficient in IFNγ signaling is not caused by the inability of the islet vasculature to present cognate antigen to autoreactive T cells through MHC class II.

## Discussion

We have studied whether IFNγ promotes migration of CD4^+^ T cells into the pancreatic islets by upregulation of MHC class II on IECs. MHC class II was rapidly upregulated in an IFNγ-dependent manner on >95% of IEC from NOD mice at the very early stages of islet infiltration and was maintained as insulitis increased. However, using a CD4^+^ T cell-mediated adoptive transfer model of autoimmune diabetes we observed that even though diabetes does not develop in *Ifn*γ*r2*-deficient recipient mice, IFNγ-induced MHC class II on IECs is not important for development of insulitis or diabetes. Costimulatory molecules such as ICOSL and CD80 (B7-1) are expressed on the vasculature (27), suggesting stimulation of T cells by endothelial cells is possible, however they are at best poor stimulators (27, 38).

IEC-MHC class II was upregulated at the same time as insulitis was first detectable in NOD mice. This occurred before MHC class II was expressed on other cell types that can upregulate MHC class II in response to inflammatory cytokines, including beta cells (36). This suggests that expression of MHC class II by IEC could be one of the earliest responses of the islet to inflammation. It is impossible to determine from our data whether MHC class II is expressed on the vasculature before, or after, the first T cells gain entry to the islet. Our data do show that it is possible for T cells to enter the islets without MHC class II expression on IEC. Our adoptive transfer experiments into chimeric mice demonstrated IEC-MHC class II is not required for diabetes to develop. In previous studies, BDC2.5 CD4^+^ T cells did not migrate into the islets in MHC cII^−/−^ mice (39). Furthermore, treating with an MHC class II-blocking antibody significantly reduced the number of T cells in each islet, and the percentage of infiltrated islets (39). However, both the knockout mouse and antibody treatment disrupts MHC class II on antigen presenting cells. It would have been ideal to use mice with EC-specific deletion of MHC class II, however, in our model we bypassed any effect on antigen presentation by reconstituting MHC class II^−/−^ mice with wildtype immune cells, thus directly assessing the role of MHC class II on non-immune cells including ECs.

Our result is surprising given the substantial evidence in the literature suggesting the requirement for MHC class I/II for transendothelial migration of T cells in models of autoimmunity and transplantation (15–19). Our findings suggest an antigen signal delivered by the ECs is not required for T cells to infiltrate the islets. It remains possible that islet macrophages could contribute to the attraction of CD4^+^ T cells by extending processes into the vascular lumen (40). Our experiments used the adoptive transfer of the well-characterized BDC2.5 T cell clone (30). The advantages of this model are: (i) that diabetes is induced rapidly, and (ii) disease induction is restricted to CD4^+^ T cells. However, BDC2.5 T cells are a monoclonal population and have a semi-activated Th1-phenotype (41), in contrast to the polyclonal, naïve T cell population that develops in the NOD mouse and potentially humans.

We confirmed previous findings that IFNγR-deficiency causes resistance to CD4^+^ T cell-mediated diabetes (42, 43). How does IFNγ promote T cell migration into the islets, if it is not induced by MHC class II upregulation on islet endothelial cells? IFNγ must be acting on a non-immune cell, and the EC is the best candidate, because beta cell specific abrogation of the IFNγR does not prevent diabetes (23). Therefore, it is likely that IFNγ induces expression of another molecule that promotes homing of only activated, diabetogenic T cells. Endothelial cell adhesion molecules (CAMs), in particular, intercellular adhesion molecule 1 (ICAM-1), vascular cell adhesion molecule 1 (VCAM-1) and mucosal vascular addressin cell adhesion molecule 1 (MadCAM-1) are upregulated in response to IFNγ (44, 45). Genetic deficiency or blocking antibodies for CAMs is generally protective (46–48), but it remains unclear whether the migration of antigen-specific CD4^+^ T cells is inhibited in these studies. All the CAM studies listed above have used global knockout mice or antibody treatments that are not specific to endothelial cells, so there is a concern that non-endothelial cell roles of CAMs might affect the results. For example, ICAM-1 plays a crucial role in immunological synapse formation (49). It would be interesting to make bone marrow chimeric mice, as we have in this study, that express CAMs on immune cells but not endothelial cells, and repeat the same BDC2.5 adoptive transfer experiments to determine whether IEC CAM expression is required for early insulitis. If adhesion molecules such as CAMs are responsible for arrest and transmigration then this is a process that does not require antigen specificity. It requires activated T cells able to transmigrate and preceding expression of IFNγ to upregulate CAMs.

In conclusion, we find MHC class II is not required on IECs to promote diabetogenic CD4^+^ T cell homing into the islets. Clearly, many questions still remain as to how IFNγ mediates T cell migration and diabetes in adoptive transfer models. Further studies, perhaps investigating the role of CAMs on ECs specifically, may reveal mechanisms as to how this occurs.

## Author contributions

NS and YZ performed experiments, analyzed data, and revised the manuscript. NS and HT designed the study and wrote the manuscript. BK, SM, and TK contributed to conception, design, and interpretation of this work and critically revised the manuscript.

### Conflict of interest statement

The authors declare that the research was conducted in the absence of any commercial or financial relationships that could be construed as a potential conflict of interest.
